# Endothelial Glycocalyx Morphology in Different Flow Regions of the Aqueous Outflow Pathway of Normal and Laser-Induced Glaucoma Monkey Eyes

**DOI:** 10.3390/cells11152452

**Published:** 2022-08-07

**Authors:** Shayna Sosnowik, David L. Swain, Neil Liu, Shan Fan, Carol B. Toris, Haiyan Gong

**Affiliations:** 1 Department of Ophthalmology, Boston University School of Medicine, Boston, MA 02118, USA; 2 Department of Anatomy and Neurobiology, Boston University School of Medicine, Boston, MA 02118, USA; 3 Department of Ophthalmology and Visual Science, University of Nebraska Medical Center, Omaha, NE 68198, USA; 4 Department of Ophthalmology and Visual Sciences, The Ohio State University, Columbus, OH 43210, USA

**Keywords:** endothelial glycocalyx, laser-induced ocular hypertension, trabecular meshwork, Schlemm’s canal, collector channel, intrascleral vein, episcleral vein, intraocular pressure, segmental outflow, monkey eye

## Abstract

Glycocalyx morphology was examined in the trabecular outflow pathway of monkey eyes with and without experimental glaucoma. Laser burns were administered along ~270 degrees of the trabecular meshwork (TM) of one eye (*n* = 6) or both eyes (*n* = 2) of each monkey until intraocular pressure remained elevated. Portions of the TM were not laser-treated. Unlasered eyes (*n* = 6) served as controls. Enucleated eyes were perfused at 15 mmHg to measure the outflow facility, perfused with fluorescein to evaluate the outflow pattern, perfusion-fixed for glycocalyx labeling, and processed for electron microscopy. Coverage and thickness of the glycocalyx were measured in the TM, Schlemm’s canal (SC), collector channels (CCs), intrascleral veins (ISVs), and episcleral veins (ESVs) in non-lasered regions and high- and low-flow regions of controls. Compared to controls, laser-treated eyes had decreased outflow facility (*p* = 0.02). Glycocalyx thickness increased from the TM to ESVs in non-lasered regions and controls (*p* < 0.05). Glycocalyx coverage was generally greater distally in non-lasered regions (*p* < 0.05). In lasered regions, TM, SC, and CCs were partly to completely obliterated, and ISVs and ESVs displayed minimal glycocalyx. Whether the glycocalyx is decreased in the trabecular outflow pathway of human glaucomatous eyes warrants investigation.

## 1. Introduction

Normal intraocular pressure (IOP) is maintained through a dynamic balance of aqueous humor production and drainage. Disruption of this balance due to increased resistance to aqueous humor outflow leads to elevation of IOP, the only modifiable risk factor for primary open-angle glaucoma (POAG) [1]. POAG is a degenerative optic neuropathy characterized by the loss of retinal ganglion cells and axons and is one of the leading causes of irreversible blindness in the United States [2]. Therefore, gaining a better understanding of how IOP and aqueous humor outflow is regulated in the trabecular outflow pathway, the primary route of egress for aqueous humor, is important [3]. To exit the eye, most of the aqueous humor passes from the anterior chamber through the trabecular outflow pathway, consisting of the following structures: trabecular meshwork (TM), Schlemm’s canal (SC), collector channels (CCs), intrascleral venous plexus, and episcleral veins (ESVs). Recent evidence suggests that the glycocalyx layer that covers the endothelial cells lining these structures of the trabecular outflow pathway may act as a mediator of trabecular outflow regulation [4,5,6].

The surface of endothelial cells is covered by a layer of proteoglycans and glycoproteins known as glycocalyx. Previous studies in the vasculature have demonstrated that the glycocalyx plays a role in mediating the alignment of endothelial cells in the direction of flow [7,8], and mechanotransduction of fluid shear stress [9,10]. One key means of mechanotransduction is via stimulation of endothelial nitric oxide synthase (eNOS), which drives the release of nitric oxide (NO), a prominent vasodilator [11,12]. Interestingly, endothelial cells within the trabecular outflow pathway appear to behave similarly to those in the vasculature. SC endothelial cells are subject to sufficient shear stress to result in the alignment of SC endothelial cells in the direction of flow [4]. In addition, transgenic mice characterized by an overexpression of eNOS have demonstrated increased outflow facility and decreased IOP compared to wild-type controls [5]. NO also leads to increased outflow facility in eyes where the TM has been removed, indicating that vasodilation of the distal outflow pathway vessels is a likely mechanism of action [6]. While the endothelial glycocalyx of the vasculature has been well studied, the glycocalyx layer within the trabecular outflow pathway has not been well explored.

A previous study characterized the glycocalyx in normal human and bovine eyes and found a variable and species-specific pattern of coverage from the TM through the CCs [13]. However, this study did not consider the segmental or non-uniform nature of trabecular outflow around the circumference of the eye. Studies in both humans [14,15,16,17] and monkeys [18,19] have demonstrated a segmental distribution of outflow in the conventional outflow pathway of normal eyes. The segmental nature of trabecular outflow is conserved to the level of the ESVs [14]. Evaluating glycocalyx morphology in regions of varying flow is of interest for several reasons. First, increasing flow leads to increased shear stress, and differences in glycocalyx dimensions can be related to local flow profile variations [20]. Secondly, studies have demonstrated that fluid shear stress can act as a stimulus for glycocalyx synthesis [20,21]. Lastly, endothelial cell changes in shape and orientation, cytoskeletal reorganization, and NO production are at least in part dependent on shear magnitude [7,10,11,12]. Because the glycocalyx acts as a mechanotransducer impacting these processes, investigating differences in the glycocalyx of different flow regions of the trabecular outflow pathway is of great interest. Given the segmental nature of trabecular outflow and its implications for shear stress variation, we hypothesize that different morphological profiles of the endothelial glycocalyx may exist in different flow regions.

While the morphology of the glycocalyx in normal human eyes has been evaluated, differences in the glycocalyx in pathologic states, such as POAG, have not been explored. Evidence suggests that the endothelial glycocalyx may play a role in both contributing to and modulating resistance. It has been demonstrated that microvessels up to 30 µm in diameter demonstrate elevated resistance compared to glass tubes of the same size, and resistance decreases with heparinase treatment [9,22]. This suggests that in vessels of smaller diameter, the presence of the glycocalyx layer may contribute to resistance. Additionally, NO has been shown to cause vasodilation and relaxation of the TM, which increases the outflow facility and decreases IOP [5]. This suggests that the glycocalyx, by stimulating NO release in response to fluid shear stress, may play a role in the regulation of IOP. Therefore, evaluating changes to the glycocalyx in an experimental monkey model of POAG, which can impact resistance via multiple mechanisms, may shed some light on the potential contributions of the glycocalyx to increased outflow resistance in POAG. 

We used a monkey model of POAG, which establishes glaucoma in monkeys via circumferential photocoagulation of the TM [23,24,25]. Not only do monkey eyes have similar anatomy to humans, but this model demonstrates ganglion cell and axon loss, optic nerve cupping, decreased outflow facility, and responds to topical ocular hypotensive medications similar to human eyes [23,24,25,26]. Alterations in segmental outflow appear similar in studies evaluating human POAG eyes and monkey eyes with laser-induced glaucoma. Previous studies in the monkey model found that fluorescent microspheres and cationic ferritin deposit in non-lasered regions of the TM, suggesting aqueous humor exits the eye through the non-lasered regions [27,28]. Interestingly, in a study by de Kater et al., wherein normal and POAG human eyes were perfused with cationic ferritin, the tracer was observed diffusely throughout the outflow pathway in normal eyes, but not in POAG eyes. Some regions of the outflow pathway in POAG eyes demonstrated tracer from the TM to the CCs while other regions showed no tracer, suggesting a pathologic process of a segmental nature resulting in preferential outflow through regions that were less affected [29]. In addition, while the ultrastructure of the laser-treated regions of the TM of the monkey eyes is not directly comparable to POAG, the outflow pathway components distal to SC that have not been lasered show some similarities to those in POAG eyes. A previous study in our laboratory demonstrated a decreased number of intrascleral veins (ISVs) distal to laser-treated regions of the TM [30]. A previous study by Dvorak-Theobald and Kirk and unpublished data from our lab evaluating the intrascleral veins distal to regions of pathologic change to the TM and SC in human POAG eyes show similar findings [31].

The purpose of our study was to investigate differences in glycocalyx morphology in regions of varying flow in normal monkey eyes as well as in a monkey model of POAG in which the glycocalyx has not been explored. This was done by examining the thickness and coverage of the glycocalyx in the trabecular outflow pathway in high- and low-flow regions of normal monkey eyes, and in laser-treated and untreated regions of monkey eyes with experimental glaucoma.

## 2. Materials and Methods

A total of 14 eyes from 8 adult female cynomolgus macaques were utilized for this study. All animals were euthanized for other studies, and eyes were enucleated for use in this study following euthanasia. One eye of six monkeys and both eyes of one monkey underwent laser photocoagulation of the TM (*n* = 8) with a Nd:YAG laser (Ophthalas 532 EyeLite Laser, Alcon Laboratories, Inc., Fort Worth, TX, USA) under isoflurane anesthesia. Contralateral eyes of six animals were not treated and served as normotensive controls. The detailed protocol for laser treatment was described previously [30]. In brief, laser photocoagulation burns were made to roughly 270 to 340 degrees of the TM. Treatments were repeated once or twice more with one month of recovery between treatments within 3 months of the first laser treatment. Persistent IOP elevation was appreciated in all but one laser-treated eye after laser treatment. The remaining TM (approximately 20–90 degrees) was not laser treated. IOPs were measured by pneumotonometry (Model 30; Reichert, Inc., Depew, NY, USA) and the mean of two measurements was recorded. If the difference between duplicate measurements was more than 2 mmHg, a third measurement was taken and the mean of the three values was recorded. IOPs were measured and recorded at two time points. Intermediate IOPs were measured 10–48 months prior to final IOPs. Final IOPs were measured 58–179 months after the final laser treatment and within 66 months of euthanasia (Table 1) [30]. Seven monkeys were used in unrelated drug trials, six of which had a washout period of ≥3 months after the last drug trial treatment, and one had a washout period of >2 months before euthanasia. Monkeys were euthanized between 58 and 196 months after their final laser treatment (mean ± SEM; 157.7 ± 13.9 months). Eyes were sent to Boston University and arrived within 24 h of enucleation. All procedures were approved by the Institutional Animal Care and Use Committee of the University of Nebraska Medical Center prior to implementation.

A detailed protocol for ocular perfusion was described previously [30]. In brief, all eyes (*n* = 8 laser-treated, and *n* = 6 controls) were perfused at 15 mmHg for 30 min to measure the outflow facility. In order to visualize segmental outflow patterns, anterior chambers of laser-treated eyes (*n* = 5) and control eyes (*n* = 3) received later in the study were exchanged and perfused with fluorescein (0.1%, Sigma-Aldrich), and ESV fluorescence was evaluated. Images of these eyes have been published previously [30]. Regions containing visible fluorescent ESVs were considered high-flow, and regions without visible ESVs were considered low-flow. While a previous study evaluated segmental flow at the level of the TM, SC, and ESVs, this study evaluated ESVs alone using fluorescein, because the fluorescent tracers that are commonly used to evaluate both proximal and distal outflow pathway structures may impact glycocalyx labeling [14]. The segmental pattern of outflow in that study was conserved proximally to distally. However, ESVs do not project directly radially from a corresponding region of TM [14]. For this reason, proximal regions of the outflow pathway adjacent to regions without fluorescent ESVs were considered low-flow, not non-flow, as minimal fluorescence in adjacent ESVs may not be a precise representation of the outflow status of proximal outflow pathway structures (TM and SC) in the same clock hour. Flow type in control eyes that did not undergo fluorescein perfusion were considered to be “undetermined” for purposes of data analysis.

Anterior chambers of all eyes were exchanged and perfusion-fixed at 15 mmHg with 1% glutaraldehyde and 4% paraformaldehyde in Dulbecco’s phosphate buffered saline containing 30 mmol/L MgCl_2_ and 0.05% (*w*/*v*) Alcian blue 8GX for 1 h. All eyes were hemisected and immersion-fixed in the same solution overnight. A previous study utilizing the same fixative demonstrated no significant differences in glycocalyx distribution in normal bovine eyes when comparing perfusion or immersion fixation [13]. One control eye was not included in the study, because the outflow facility was abnormally high during perfusion, potentially due to a leak in the globe.

A comprehensive protocol for tissue dissection and embedding was described in a previous study [30]. In brief, anterior segments were dissected into ~30 radial wedges, which were processed and embedded for electron microscopy. Semi-thin (2 µm) sections were cut and stained with 1% toluidine blue to identify the location of outflow pathway structures. Ultra-thin (70–80 nm) sections were cut and examined via transmission electron microscopy (JEOL JEM-1400 Flash, Peabody, MA, USA). Electron micrographs (≥30 images per eye) were taken randomly at different locations (TM, inner wall of SC, CC, ISV, ESV) of the aqueous outflow pathway and pooled for each location by flow region, including high-flow, low-flow, and undetermined-flow regions of control eyes, and non-lasered regions of laser-treated eyes, to evaluate thickness and percent coverage of the glycocalyx (≥14 measurements per location for each flow region). Percent coverage of glycocalyx ((length of glycocalyx covering endothelial surface/total length of endothelial surface)*100) was determined by the intensity plot profile of the glycocalyx adjacent and parallel to the endothelial cell membrane using ImageJ (Version 1.53g, National Institutes of Health, Bethesda, MD, USA). A preset intensity was used to differentiate glycocalyx from the background. Thickness was measured as a perpendicular line from the cell membrane at the base of the glycocalyx strand or bundle to the termination of the strand or bundle. The mean thickness of the glycocalyx was determined from three sites within each image where the glycocalyx was intact and measurable.

IOP and outflow facility data are listed as the median and interquartile range (IQR). All other data are listed as the mean and standard error of the mean (SEM). IOP and outflow facility data were compared between control and laser-treated eyes using Wilcoxon rank-sum tests. Both coverage and thickness were evaluated within individual outflow pathway locations between non-lasered regions of laser-treated eyes and control regions (high-flow, low-flow, and undetermined-flow), as well as between high- and low-flow control regions. While a minority of data sets, assessed using histograms, revealed non-normal distributions or unequal within-group variances, a sensitivity analysis wherein the tests were run with and without the problematic data sets, did not show significantly different results. For this reason, all data sets were included, and ANOVA tests followed by post hoc Tukey honest significant difference (HSD) tests were used to compare the data sets. Coverage and thickness were also evaluated within each individual flow-type region between all outflow locations via ANOVA tests followed by post hoc Tukey HSD tests. A subset of all measurements was repeated after at least one week by the same individual (S.S.) or a second individual (N.L.), and the differences between the original measurements and the repeated subsets were ≤10%. 

## 3. Results

### 3.1. Intraocular Pressure

Median final IOP was greater in laser-treated eyes (28.7 mmHg, IQR: 24.3–31.1; *n* = 8) than control eyes (23.2 mmHg, IQR: 22.0–26.7; *n* = 6); however, this difference was not statistically significant (*p* = 0.30). Median intermediate IOP was significantly greater in laser-treated eyes (34.5 mmHg, IQR: 29.5–35.2; *n* = 7), then control eyes (25.1 mmHg, IQR: 23.3–27.8; *n* = 4; *p* = 0.04) (Table 1).

### 3.2. Outflow Facility

Median outflow facility was significantly decreased in laser-treated eyes (0.08 μL/min/mmHg, IQR: 0.03–0.17; *n* = 8), compared to control eyes (0.32, IQR: 0.29–0.44; *n* = 6; *p* = 0.02).

### 3.3. Glycocalyx Morphology

**Figure 1:**
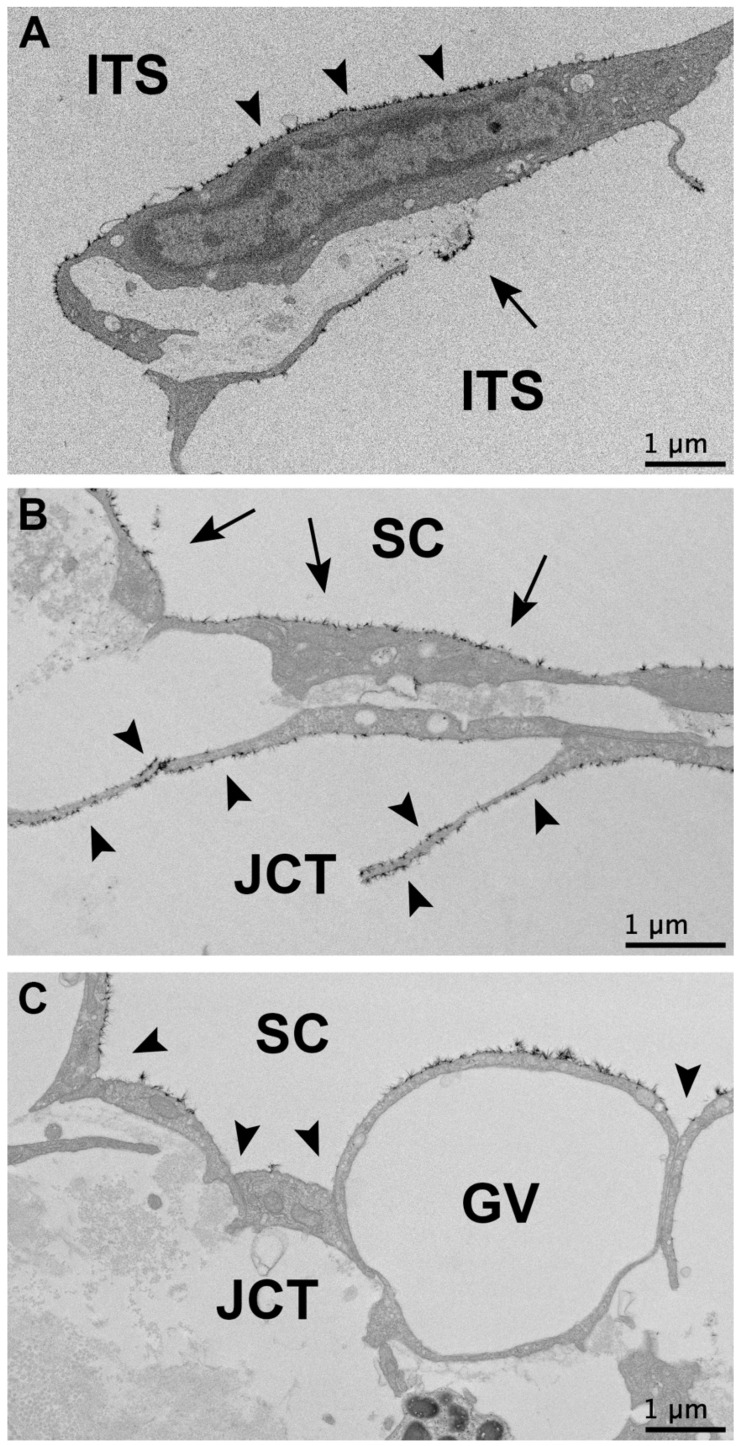
Non-uniform glycocalyx coverage along the trabecular outflow pathway. (**A**) A representative example of glycocalyx coverage on the trabecular beams in an undetermined-flow region of a control eye. Arrowheads indicate glycocalyx covering the trabecular cell membrane. The arrow indicates glycocalyx overlying the exposed basement membrane in a region of the trabecular beam without endothelial cell coverage. (**B**) Non-uniform glycocalyx coverage of juxtacanalicular connective tissue (JCT) cells. Arrows indicate glycocalyx covering the inner wall of Schlemm’s canal (SC). Arrowheads indicate glycocalyx coverage on the JCT cells. Shown in an undetermined-flow region of a control eye. (**C**) Inner wall endothelial cell surfaces protruding into lumens displayed more glycocalyx coverage than recessed regions (arrowheads) in Schlemm’s canal (SC) and collector channels (CCs). Shown in SC in a low-flow region of a control monkey eye. No glycocalyx coverage was seen along the inner cellular membrane of the giant vacuole (GV). ITS: intertrabecular space.

**Figure 2:**
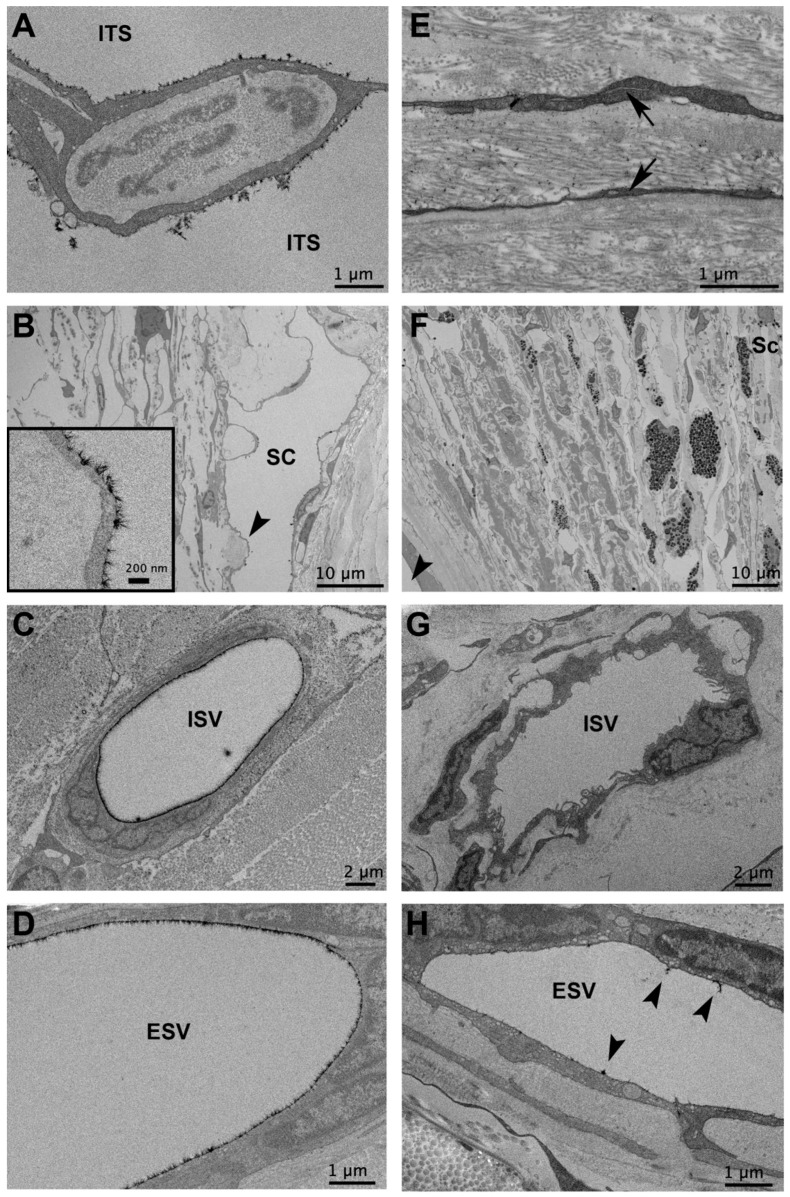
Differences in morphology and glycocalyx coverage in control and laser-treated outflow pathway locations. In control eyes, trabecular beams were separated by notable intertrabecular spaces (ITSs; (**A**)), Schlemm’s canal (SC) was patent (**B**) with glycocalyx coverage, (arrowhead indicates region shown at high magnification in inset image), and both intrascleral veins (ISVs; (**C**)) and episcleral veins (ESVs; (**D**)) displayed significant glycocalyx coverage. In contrast, in laser-treated regions, ITSs were collapsed (arrows; (**E**)), and SC was partially to completely obliterated (**F**). The arrowhead in (**F**) indicates the anterior chamber. In addition, ISVs (**G**) and ESVs (**H**) in laser-treated regions displayed minimal to absent glycocalyx. The arrowheads in (**H**) indicate small clusters of the glycocalyx in an ESV. Sc: sclera.

In all flow regions of control eyes and non-lasered regions of laser-treated eyes, a non-uniform glycocalyx was found covering the trabecular beams, and juxtacanalicular connective tissue (JCT) cells, the inner walls of SC, and the walls of CCs, ISVs, and ESVs. The trabecular beams frequently displayed glycocalyx coverage on the trabecular cell surface, as well as on the exposed basement membrane when the trabecular endothelial cell did not fully cover the beam (Figure 1A) in undetermined, high- and low-flow regions of control eyes, and non-lasered regions of laser-treated eyes. Glycocalyx coverage was observed on the surface of JCT cells (Figure 1B). Glycocalyx coverage on the inner wall of SC and CCs was often observed to be more uniform in regions where the endothelial cells protruded into the lumen of SC and CCs and were less uniform in more recessed areas (Figure 1C). ISVs and ESVs often displayed a prominent glycocalyx layer. In contrast to control and non-lasered regions, laser-treated regions displayed significant alterations in the morphology of outflow pathway structures and decreased glycocalyx coverage. Intertrabecular spaces were frequently collapsed, trabecular beams were damaged, and SC and CCs were partially to completely obliterated. Outflow pathway locations from the TM to ESVs in laser-treated regions displayed minimal to absent glycocalyx and were thus not included in measurements of glycocalyx coverage and thickness (Figure 2).

#### 3.3.1. Glycocalyx Coverage

**Figure 3:**
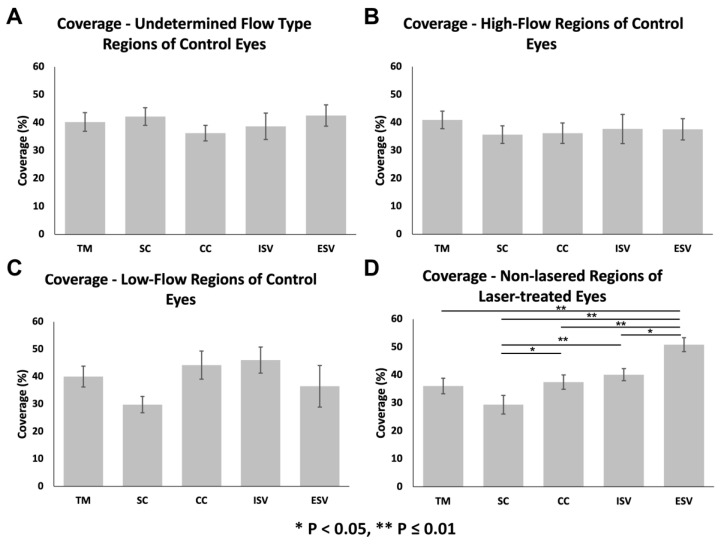
Glycocalyx coverage along the different locations of the outflow pathway in control eyes and non-lasered regions of laser-treated eyes. (**A**–**C**): Glycocalyx coverage did not differ significantly between outflow pathway locations in undetermined-flow type (ANOVA, *F* = 0.42, *p* = 0.86), and high-flow regions (ANOVA, *F* = 1.42, *p* = 0.21) of control eyes. While coverage was significantly different across all outflow locations within low-flow regions of the control eyes (ANOVA, *F* = 2.24, *p* = 0.04), no significant differences were noted for any pairwise comparison of locations. (**D**): Non-lasered regions of the laser-treated eyes displayed a significant increasing trend in glycocalyx coverage proximally to distally along the conventional outflow pathway (ANOVA, *F* = 11.57, *p* ≤ 0.01). Error bars: SEM. * *p* < 0.05, ** *p* ≤ 0.01.

**Figure 4:**
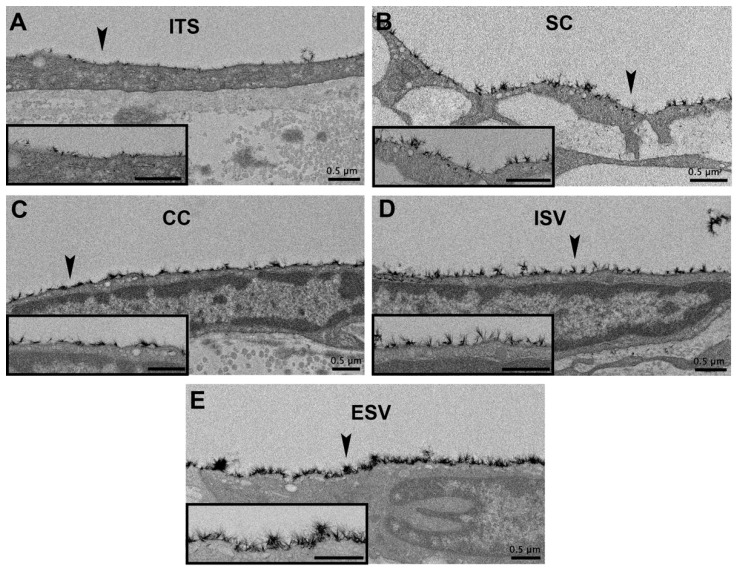
Increasing glycocalyx coverage and thickness along the outflow pathway in non-lasered regions of laser-treated eyes. Arrowheads in (**A**–**E**) highlight portions of glycocalyx evaluated at high magnification in the inset images of each respective panel. In evaluating non-lasered regions in the (**A**) trabecular meshwork (TM), (**B**) Schlemm’s canal (SC), (**C**) collector channels (CCs), (**D**) intrascleral veins (ISVs), and (**E**) episcleral veins (ESVs), both glycocalyx coverage and thickness displayed a general increasing trend proximally to distally along the outflow pathway. ITS: intertrabecular space.

The percentage of glycocalyx coverage of different outflow pathway locations was not significantly different in undetermined-flow (ANOVA, *F* = 0.42, *p* = 0.86; Figure 3A), and high-flow regions (ANOVA, *F* = 1.42, *p* = 0.21; Figure 3B) of control eyes. The percentage of glycocalyx coverage of different outflow pathway locations was significantly different across all locations in low-flow regions of control eyes (ANOVA *F* = 2.24, *p* = 0.04; Figure 3C); however, no specific pairwise comparisons were significantly different. In non-lasered regions of laser-treated eyes, outflow pathway locations had significantly different percentage coverages of the glycocalyx. Generally, more distal structures, such as ESVs, had larger percentage coverages than more proximal structures, (ANOVA, *F* = 11.57, *p* ≤ 0.01; Figure 3D and Figure 4). Specifically, glycocalyx coverage of the TM (36.1 ± 2.8%; *n* = 44) was significantly lower than ESVs (50.9 ± 2.5%; *n* = 46; *p* ≤ 0.01); coverage of SC (29.4 ± 3.4%; *n* = 43) was significantly lower than CCs (37.4 ± 2.6%; *n* = 46; *p* = 0.04), ISVs (40.1 ± 2.1%; *n* = 38 *p* ≤ 0.01), and ESVs (*p* ≤ 0.01); coverage of CCs was significantly lower than ESVs (*p* ≤ 0.01); coverage of ISVs was significantly lower than ESVs (*p* = 0.05). Coverages of all other pairs of outflow locations within non-lasered regions were not significantly different. Coverage of the TM was not significantly different from SC (*p* = 0.13), CCs (*p* = 0.99), or ISVs (*p* = 0.67), and coverage of CCs was not significantly different than ISVs (*p* = 0.90) (Figure 3D).

**Figure 5:**
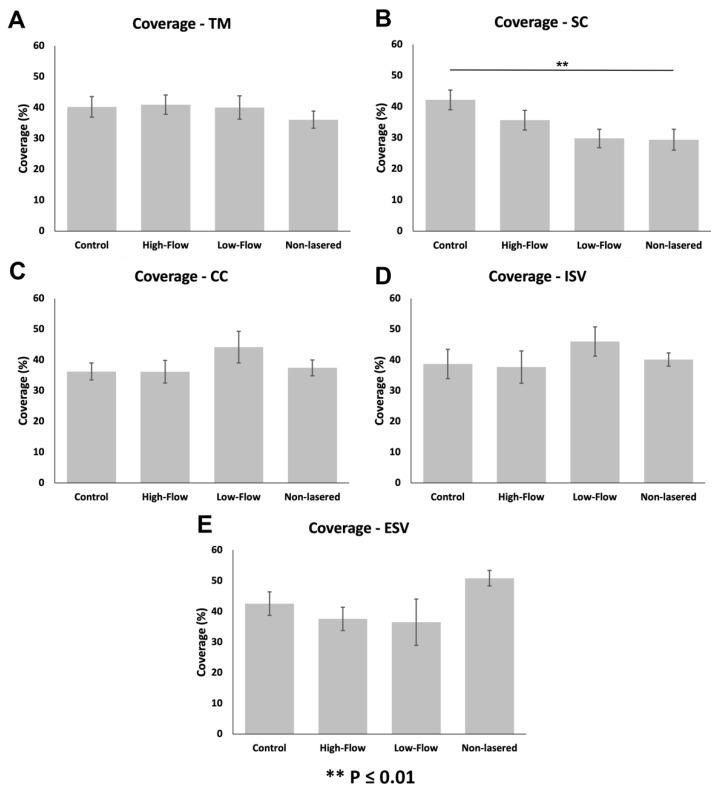
Glycocalyx coverage between different flow regions within various outflow pathway locations (**A**): Coverages between flow regions of the TM were not significantly different (ANOVA, *F* = 0.86, *p* = 0.47). (**B**): Coverage was significantly lower in SC of non-lasered regions (29.4 ± 3.4%; *n* = 43) than undetermined-flow regions of control eyes (42.2 ± 3.2%; *n* = 30; *p* ≤ 0.01). No significant difference in coverage in SC was noted between any other flow regions (*p* > 0.05). (**C**): Coverages between flow regions of CCs were not significantly different (ANOVA, *F* = 1.01, *p* = 0.39). (**D**): Coverages between flow regions of ISVs were not significantly different (ANOVA, *F* = 0.91, *p* = 0.44). (**E**): Glycocalyx coverage across all different flow regions was significantly different in ESVs (ANOVA, *F* = 3.54, *p* = 0.02); however, pairwise comparisons revealed no significant difference in coverage. Error bars: SEM. ** *p* ≤ 0.01.

When comparing coverages between flow regions within individual outflow pathway locations, coverages of SC (ANOVA, *F* = 4.77, *p* ≤ 0.01; Figure 5B) were significantly different between flow regions. Coverage was significantly lower in the SC of non-lasered regions (29.4 ± 3.4%; *n* = 43) than undetermined-flow regions of control eyes (42.2 ± 3.2%; *n* = 30; *p* ≤ 0.01). No significant difference in coverage in SC was noted between non-lasered regions and low-flow (29.8 ± 3.0%; *n* = 28; *p* = 0.97) or high-flow regions (35.7 ± 3.2%; *n* = 30; *p* = 0.26), or between high-flow and low-flow regions of control eyes (*p* = 0.59). The percentage of glycocalyx coverage across all different flow regions was significantly different in ESVs (ANOVA, F = 3.54, *p* = 0.02; Figure 5E); however, pairwise comparisons revealed no significant difference in coverage in ESVs between non-lasered regions (50.9 ± 2.5%; *n* = 46) of laser-treated eyes and low-flow regions (36.5 ± 7.6%; *n* = 14; *p* = 0.05), high-flow regions (37.6 ± 3.8%; *n* = 20; *p* = 0.06), or undetermined-flow regions (42.6 ± 3.8%; *n* = 21; *p* = 0.38) of control eyes, or between high-flow and low-flow regions (*p* = 0.98) of control eyes. Coverages between flow regions of the TM (ANOVA, *F* = 0.86, *p* = 0.47; Figure 5A), CCs (ANOVA, *F* = 1.01, *p* = 0.39; Figure 5C), and ISVs (ANOVA, *F* = 0.91, *p* = 0.44; Figure 5D) were not significantly different.

#### 3.3.2. Glycocalyx Thickness

**Figure 6:**
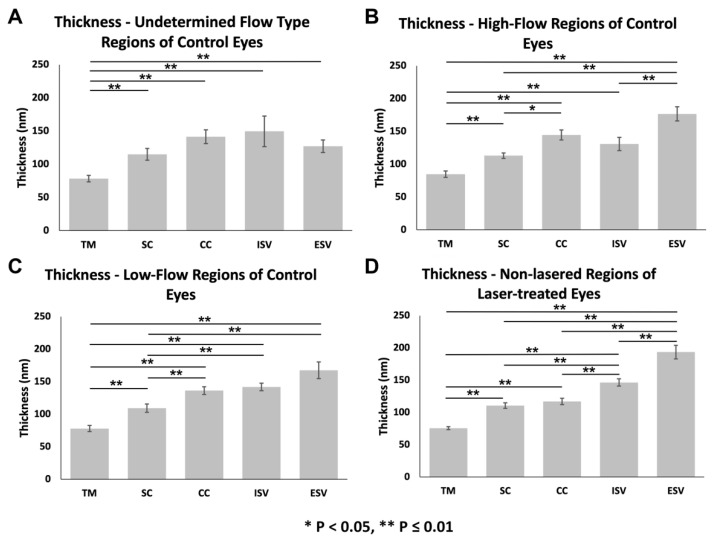
Glycocalyx thickness along the different locations of the outflow pathway by flow region. (**A**) Undetermined-flow type regions of control eyes (ANOVA, *F* = 13.99, *p* ≤ 0.01), (**B**) high-flow regions of control eyes (ANOVA, *F* = 26.45, *p* ≤ 0.01), (**C**) low-flow regions of control eyes (ANOVA, *F* = 31.57, *p* ≤ 0.01), and (**D**) non-lasered regions of laser-treated eyes (ANOVA, *F* = 117.81, *p* ≤ 0.01) displayed a significant increasing trend in glycocalyx thickness proximally to distally along the conventional outflow pathway. Error bars: SEM. * *p* < 0.05, ** *p* ≤ 0.01.

In general, thickness increased proximally to distally along the outflow pathway across all flow-type regions evaluated (Figure 6), including undetermined-flow regions of controls (ANOVA, *F* = 13.99, *p* ≤ 0.01; Figure 6A), high-flow regions of controls (ANOVA, *F* = 26.45, *p* ≤ 0.01; Figure 6B), low-flow regions of controls (ANOVA, *F* = 31.57, *p* ≤ 0.01; Figure 6C), and non-lasered regions of lasered eyes (ANOVA, *F* = 117.81, *p* ≤ 0.01; Figure 6D). 

In undetermined-flow regions of control eyes, the glycocalyx was significantly thinner in the TM (78.3 ± 4.8 nm; *n* = 30) than SC (114.9 ± 8.9 nm; *n* = 30; *p* ≤ 0.01), CCs (141.4 ± 10.5 nm; *n* = 30; *p* ≤ 0.01), ISVs (149.5 ± 23.1 nm; *n* = 25; *p* ≤ 0.01), and ESVs (127.0 ± 9.5 nm; *n* = 25; *p* ≤ 0.01) (Figure 6A). Glycocalyx thickness was not significantly different between the following: SC and CCs (*p* = 0.30), ISVs (*p* = 0.83), or ESVs (*p* = 0.84); between CCs and ISVs (*p* = 0.99) or ESVs (*p* = 0.99); between ISVs and ESVs (*p* = 1.0) (Figure 6A). 

In high-flow regions of control eyes, the glycocalyx was significantly thinner in the TM (84.8 ± 4.7 nm; *n* = 30) than SC (113.0 ± 4.1 nm; *n* = 30; *p* ≤ 0.01), CCs (144.6 ± 7.5 nm; *n* = 28; *p* ≤ 0.01), ISVs (130.9 ± 10.1 nm; *n* = 25; *p* ≤ 0.01), and ESVs (176.7 ± 10.8 nm; *n* = 20; *p* ≤ 0.01) (Figure 6B). In addition, the glycocalyx was significantly thinner in SC than CCs (*p* < 0.05) and in both SC and ISVs compared to ESVs (both *p* ≤ 0.01). Glycocalyx thickness was not significantly different between SC and ISVs (*p* = 0.85) or between CCs and ISVs (*p* = 0.70) or ESVs (*p* = 0.18) (Figure 6B). 

In low-flow regions of control eyes, the glycocalyx was significantly thinner in the TM (77.9 ± 4.7 nm; *n* = 28) compared to SC (109.1 ± 6.4 nm; *n* = 30; *p* ≤ 0.01), CCs (136.3 ± 5.9 nm; *n* = 30; *p* ≤ 0.01), ISVs (141.9 ± 5.8 nm; *n* = 15; *p* ≤ 0.01), and ESVs (167.5 ± 12.7 nm; *n* = 15; *p* ≤ 0.01) (Figure 6C). The glycocalyx was also significantly thinner in SC than CCs (*p* ≤ 0.01), ISVs (*p* ≤ 0.01), and ESVs (*p* ≤ 0.01). Glycocalyx thickness was not significantly different between CCs and ISVs (*p* = 0.99) or ESVs (*p* = 0.23), or between ISVs and ESVs (*p* = 0.78) (Figure 6C). 

Lastly, in non-lasered regions of laser-treated eyes, the glycocalyx was significantly thinner in the TM (75.7 ± 2.3 nm; *n* = 54) compared to SC (110.7 ± 4.3 nm; *n* = 43; *p* ≤ 0.01), CCs (117.0 ± 4.8 nm; *n* = 48; *p* ≤ 0.01), ISVs (146.4 ± 5.7 nm; *n* = 40; *p* ≤ 0.01), and ESVs (193.4 ± 10.4 nm; *n* = 45; *p* ≤ 0.01) (Figure 6D). The glycocalyx was also significantly thinner in SC and CCs than both ISVs (both *p* ≤ 0.01) and ESVs (both *p* ≤ 0.01), and in ISVs than ESVs (*p* ≤ 0.01). Glycocalyx thickness was not significantly different between SC and CCs (*p* = 0.80) in non-lasered regions of laser-treated eyes (Figure 6D). 

**Figure 7:**
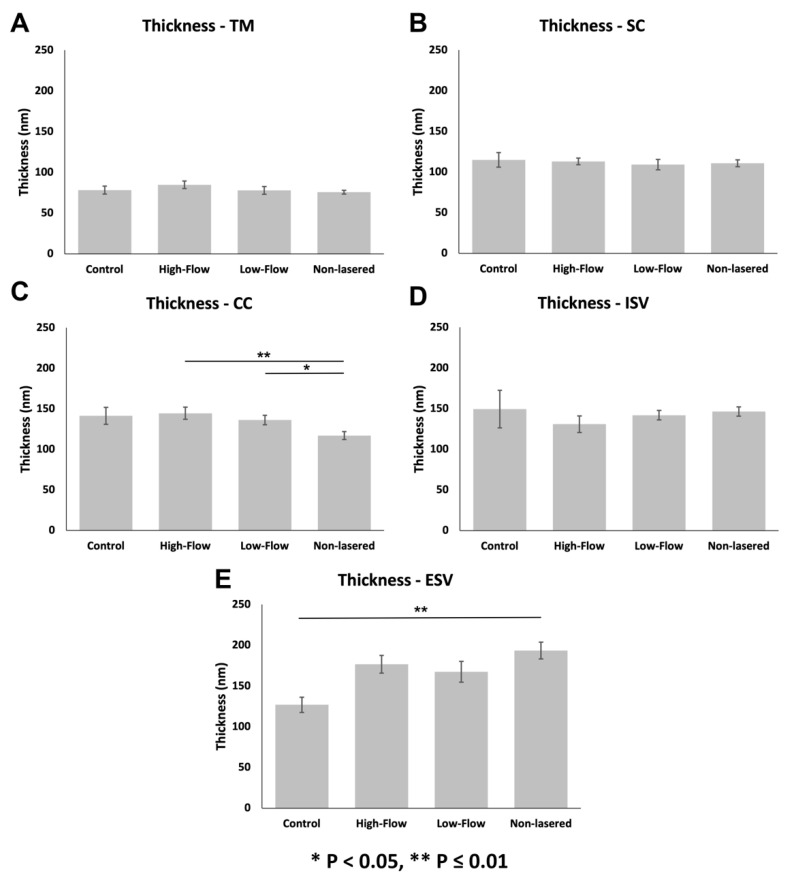
Glycocalyx thickness between different flow regions within various outflow pathway locations (**A**): Thicknesses between flow regions of the TM were not significantly different (ANOVA, *F* = 1.03, *p* = 0.38) (**B**): Thicknesses between flow regions of SC were not significantly different (ANOVA, *F* = 0.53, *p* = 0.66). (**C**): Glycocalyx thickness in CCs was significantly lower in non-lasered regions than high-flow regions (*p* ≤ 0.01) and low-flow regions (*p* = 0.04) of controls. (**D**): Glycocalyx thickness across all different flow regions was significantly different in ISVs (ANOVA, *F* = 2.91, *p* = 0.04); however, pairwise comparisons revealed no significant difference in coverage. (**E**): Glycocalyx thickness in the ESVs was significantly greater in non-lasered regions compared to undetermined-flow regions of control eyes (*p* ≤ 0.01). Error bars: SEM. * *p* < 0.05, ** *p* ≤ 0.01.

When comparing thicknesses of individual outflow pathway locations between flow-type regions, the thickness of CCs (ANOVA, *F* = 4.56, *p* ≤ 0.01; Figure 7C) and ESVs (ANOVA, *F* = 13.29, *p* ≤ 0.01; Figure 7E) between regions were significantly different. Specifically, glycocalyx thickness in CCs was significantly lower in non-lasered regions than in high-flow regions (*p* ≤ 0.01) and low-flow regions (*p* = 0.04) of controls. No significant differences in thickness in CCs were observed between high-flow and low-flow regions (*p* = 0.92) of controls or between non-lasered regions of laser-treated eyes and undetermined-flow regions of control eyes (*p* = 0.22). Glycocalyx thickness in the ESVs was significantly greater in non-lasered regions compared to undetermined-flow regions of control eyes (*p* ≤ 0.01). No significant differences in thickness in ESVs were noted between non-lasered regions and low-flow (*p* = 0.57) and high-flow regions (*p* = 0.91) of controls or between low-flow and high-flow regions (*p* = 0.93) of controls. Glycocalyx thickness across all flow regions was significantly different in ISVs (ANOVA, *F* = 2.91, *p* = 0.04; Figure 7D); however, no specific pairwise comparisons were significantly different. Thicknesses between regions of the TM (ANOVA, *F* = 1.03, *p* = 0.38; Figure 7A) and SC (ANOVA, *F* = 0.53, *p* = 0.66; Figure 7B) were not significantly different between flow-type regions.

#### 3.3.3. Glycocalyx Associated with Giant Vacuoles and I-Pores

The endothelial cells of SC are connected to each other by tight junctions [32,33]. Aqueous humor passes through the TM and into SC via two routes: (1) through intercellular openings, or pores, that form between adjacent inner wall endothelial cells (B-pores); (2) through intracellular pores (I-pores), which are usually associated with a giant vacuole (GV) [34,35,36,37]. GVs are aqueous humor-filled outpouchings, which appear to form due to the pressure drop between the anterior chamber and episcleral veins [36,38,39]. Previous studies have found that approximately 14.5% of GVs have one or more I-pores, and these GVs with I-pores are larger in size with a thinner cellular lining around the GV [37]. Resistance to aqueous humor outflow is believed to be modulated by a funneling effect of the pores in the inner wall endothelium [40].

**Figure 8:**
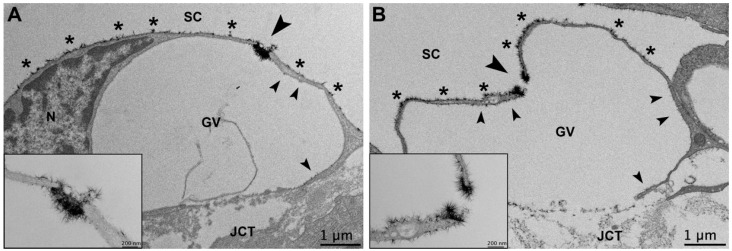
I-pores of giant vacuoles (GVs) filled or unfilled with glycocalyx. (**A**) An example of an I-pore in a GV completely filled with glycocalyx. The large arrowhead indicates the glycocalyx filling an I-pore shown at high magnification in the lower left corner of the panel. The asterisks indicate the glycocalyx on the luminal surface of Schlemm’s canal (SC), and the small arrowheads indicate the glycocalyx on the inner lining of the GV. (**B**) An example of an I-pore of a GV with increased glycocalyx around the edge of the pore, but an unfilled center. The large arrowhead indicates the glycocalyx incompletely filling an I-pore shown at high magnification in the lower left corner of the panel. The asterisks indicate glycocalyx on the luminal surface of SC and the small arrowheads indicate the glycocalyx along the inner lining of the GV. JCT: juxtacanalicular connective tissue; N: inner wall endothelial cell nucleus.

The overall glycocalyx distribution inside the cellular lining and on the luminal surface of GVs was significantly different based on whether they contained an I-pore or not (Chi-square test for independence, χ^2^ = 8.13, *p* ≤ 0.01). The majority (39/60, 65.0%) of GVs without an associated I-pore contained glycocalyx lining their apical surface only. The remainder (21/60, 35%) contained sparse glycocalyx on the inner lining of the GV or associated with the matrix of the basal opening when present in addition to on the apical surface. All five GVs with I-pores (5/5, 100%) displayed glycocalyx lining their apical surfaces, sparse to moderate glycocalyx lining the inner surfaces of the GVs, particularly near the pores and basal openings when present, and an increased amount of glycocalyx near or filling the opening of the pore (Figure 8). It is possible that the GVs containing glycocalyx on their inner lining that did not contain I-pores simply did not have I-pores in the sections examined, and may have had pores in adjacent unimaged sections.

## 4. Discussion

Our evaluation of endothelial glycocalyx morphology in this study elaborated on the patterns of distribution in normal monkey eyes, particularly the effects of segmental outflow, and examined for the first time the glycocalyx patterns in a monkey model of POAG. Morphological changes and trends of the glycocalyx noted in this study included: (1) increasing glycocalyx thickness proximally to distally along the outflow pathway in all flow-type regions of control eyes and in non-lasered regions of laser-treated eyes, (2) no significant differences in thickness or coverage between high- and low-flow regions of control eyes, and (3) quantitative and/or qualitative alterations in glycocalyx coverage in both non-lasered regions and laser-treated regions relative to normal eyes. These changes may have implications regarding endothelial glycocalyx function and IOP regulation in both normal and pathologic states.

In all regions of controls and in non-lasered regions of laser-treated eyes, an increase in glycocalyx thickness proximally to distally along the aqueous outflow pathway was observed in monkey eyes. A similar trend was documented in a previous study evaluating glycocalyx thickness in human eyes, with a significant increase in glycocalyx thickness from the TM to SC and from SC to CCs [13]. One possible explanation for this trend is the transition to unperturbed flow proximally to distally along the outflow pathway. A previous study has documented that vascular endothelial glycocalyx thickness in mice is significantly lower in the left internal carotid sinus region compared to the left common carotid artery surface, and it was hypothesized that the turbulent flow pattern near arterial bifurcations may alter glycocalyx composition [41]. Turbulent blood flow has been associated with stenotic lesions within the vasculature as well [42]. Aqueous humor enters the conventional outflow pathway through a meshwork of trabecular beams, which may conceivably act as branch points, dividing aqueous humor flow as it moves towards the JCT. Then, aqueous humor is thought to be funneled through micron-sized pores to enter into SC [43]. These unique anatomical features of the proximal outflow pathway may have a similar effect on aqueous humor dynamics to those seen in the vasculature, resulting in more disruptions in flow. Aqueous humor flow would be expected to have more unobstructed laminar flow as aqueous humor progresses from SC and CCs to the intrascleral and episcleral veins [44]. The possible transition in flow dynamics proximally to distally along the conventional outflow pathway may explain why glycocalyx thickness generally appears to increase progressively from the TM to the ESVs.

A second possible explanation for the trend of increasing glycocalyx proximally to distally is the transition from lumens containing aqueous humor to ESVs containing blood. Adamson and Clough demonstrated that the interaction of the glycocalyx with proteins can impact their structure [45]. Perfusion of frog mesenteric capillaries with cationized ferritin followed by either frog plasma or bovine serum albumin significantly lifted the layer of ferritin above the endothelial surface, suggesting an interaction of the glycocalyx with proteins resulting in altered glycocalyx organization [45]. Given the increased protein content of blood compared to aqueous humor, this may explain the increased thickness of the glycocalyx in the ESVs. 

Another interesting finding in this study in both control eyes and non-lasered regions of laser-treated eyes was the concentration of glycocalyx partially or completely filling GV-associated I-pores in SC. Yang et al. noted similar findings in I-pores of SC and the aqueous plexus in human and bovine eyes, respectively [13]. Pore-like structures within the vasculature appear to demonstrate similar findings. A study by Rostgaard and Qvortrup evaluated fenestrated capillaries in the small intestine, stomach, and peritubular region of the kidneys of rats and found that fenestrae contain bush-like filamentous sieve plugs composed of negatively charged proteoglycans. These structures were hypothesized to regulate the diffusion of water and solutes on the basis of their structure, location, and charge [46]. The glycocalyx has been hypothesized to modulate resistance in microvessels up to 30 µm in diameter [9,22]. Thus, the micron-sized I-pores within the apical surface of GVs may contribute significantly to outflow resistance depending on whether “plugs” of glycocalyx are in place [13]. Unfortunately, we did not find a sufficient sample size of I-pores to compare glycocalyx composition within I-pores between regions of varying flow in our study.

One goal of this study was to examine differences in normal glycocalyx configurations based on varying flow profiles. Previous studies in the vasculature have demonstrated that glycocalyx distribution, dimensions, and synthesis are impacted by variations in flow profiles and associated shear stress [20,21]. As mathematical modeling of aqueous humor flow in SC has demonstrated that shear stress within SC can reach levels seen in the arterial system and is variable circumferentially around SC, we hypothesized that the segmental nature of aqueous outflow may result in varying glycocalyx morphology in varying flow regions [4]. However, while individual flow regions of control eyes displayed differences in glycocalyx morphology by outflow pathway location, no significant differences in either glycocalyx coverage or thickness were noted between high- and low-flow regions of normal monkey eyes in this study. There are several possible explanations for this finding. While six control eyes were utilized in this study, only three were perfused with fluorescein to allow for an evaluation of the impact of segmental outflow on glycocalyx morphology. A larger sample size may be necessary to shed light on potential differences in glycocalyx distribution in regions of differing flow. Additionally, evaluating segmental flow in more proximal outflow pathway regions in addition to ESVs to better pinpoint exact locations where shear forces would be expected to differ may be helpful. Lastly, computed wall shear stresses in SC have demonstrated maximal flow near CC ostia and significantly increased shear stresses with decreases in the height of SC [4]. Evaluation of glycocalyx morphology in SC as a function of SC height and proximity to CC ostia may be of interest in the future.

The second goal of this study was to evaluate glycocalyx patterns in a model of POAG. Degradation of the endothelial glycocalyx has been implicated in the pathophysiology of multiple vascular diseases, such as atherosclerosis, stroke, hypertension, kidney disease, and sepsis [47]. However, changes to the endothelial glycocalyx in diseases involving the aqueous outflow pathway, such as glaucoma, have not been evaluated. In this study, we evaluated the glycocalyx in a model of POAG using laser treatment of the TM in monkeys. Lasered regions of laser-treated eyes displayed vastly altered morphology compared to non-lasered regions and control eyes (Figure 2). The trabecular beams appeared to collapse against one another, and SC and CCs were partially to completely obliterated. In addition, minimal glycocalyx labeling was noted not only in regions directly affected by laser treatment, such as the TM, but also as far distally as the ESVs. One possible explanation for the negligible glycocalyx labeling in all outflow pathway locations of laser-treated regions is the inability of perfusate containing Alcian blue to reach those locations. Tracer studies in this model have suggested minimal outflow through laser-treated regions of the TM [27,28]. Interestingly, as tracer studies in human POAG eyes seem to demonstrate similar regions of negligible aqueous outflow compared to normal eyes, we might expect similar findings [29]. This may be compounded by the fact that shear stress acts as a stimulus for glycocalyx synthesis and, with minimal flow through a damaged TM, the balance of glycocalyx shedding and synthesis may be disrupted [20,21]. The majority of GVs without I-pores in this study did not display glycocalyx on their inner lining, whereas all GVs with I-pores demonstrated glycocalyx apically and interiorly, which supports a relationship between shear stress and glycocalyx synthesis. While perfusion fixation may have been hampered by morphological changes in the proximal outflow pathway, a previous study using similar methods indicated that immersion fixation provides similar labeling to perfusion fixation [13]. As immersion fixation would be expected to label distal elements of the outflow pathway when fixative diffuses across the sclera, all samples in this study were both perfusion- and immersion-fixed.

A second possibility for the lack of glycocalyx labeling in lasered regions of laser-treated eyes is that the decrease in glycocalyx in these regions is a result of chronic, low-grade inflammation following laser treatment. Exposure to chronic inflammation may explain the decreased coverage in more proximal regions of the outflow pathway in non-lasered regions, compared to other flow-type regions or more distal non-lasered outflow pathway locations. Acute exposure to various inflammatory mediators, such as tumor necrosis factor-alpha (TNF-α), matrix metalloproteinases (MMPs), and thrombin is associated with increased glycocalyx shedding [48,49,50,51,52]. However, the impact of more chronic inflammation on the glycocalyx remains unclear. These findings are of interest because some studies evaluating TM and aqueous humor samples from human POAG eyes have observed increased levels of various inflammatory mediators, including TNF-α and various MMPs [53,54,55,56,57]. The endothelial glycocalyx within the aqueous outflow pathway may play a role in the mechanotransduction of fluid shear stress via the mediation of NO release similar to that in the vasculature [5,11,12]. Nitric oxide has been demonstrated or implicated in increasing outflow facility via relaxation of the TM, decreasing TM and SC endothelial cell volume [58,59,60]. NO-induced vasodilation of distal outflow pathway vessels is a likely mechanism of action as well [6]. Given the potential role of the glycocalyx in the outflow pathway as a mechanosensor that contributes to NO release, compromise of the glycocalyx due to the increased concentration of these sheddases may have significant effects on aqueous outflow resistance and IOP regulation. 

One limitation of this study is the inability to correlate findings in the proximal outflow pathway of our model with human POAG eyes due to the laser treatment. However, as previously mentioned, both the lasered monkey eyes and human POAG eyes demonstrate evidence of negligible outflow through affected regions of the TM, shunting of aqueous humor to unaffected regions of the proximal outflow pathway, and changes to ISVs in the outflow pathway distal to affected proximal outflow pathway locations [27,28,29,30]. Due to these parallels, we believe the lasered monkey model is helpful for evaluating changes in glycocalyx distribution following segmental outflow changes due to proximal outflow pathway pathology (i.e., laser treatment in this model). In summary, this study has further elucidated the normal structure of the glycocalyx in multiple outflow pathway locations, as well as between regions of varying flow in normal monkey eyes. Anatomical variations between outflow pathway locations appear to play a role in glycocalyx morphology. In addition, whether morphological changes of the endothelial glycocalyx, specifically reductions in coverage, may have impacted outflow resistance and IOP regulation in addition to laser damage to the TM in our POAG model requires further investigation. Investigation of whether the glycocalyx is decreased in human POAG eyes is warranted. 

## Figures and Tables

**Table 1:** Intermediate and final IOPs in control and laser-treated monkey eyes.

	Control		Laser-Treated		
Monkey	Intermediate IOP (mmHg)	Final IOP (mmHg)	Intermediate IOP (mmHg)	Final IOP (mmHg)	Age at Final IOP (Years)
1	N/A	22.0	N/A	20.0	N/A
2	26.5	22.0	35.8	31.0	15
3	23.7	29.9	30.0	31.5	17
4	31.5	19.6	64.8	29.8	18
5	22.0	27.5	28.4	31.3	18
6/OS			28.9	25.7	15
6/OD			34.5	27.5	15
7			34.6	19.3	18
8	N/A	24.3			19

## Data Availability

The raw data are readily available upon reasonable request from the corresponding author.
